# Skeletal and Respiratory Muscle Dysfunctions in Pulmonary Arterial Hypertension

**DOI:** 10.3390/jcm9020410

**Published:** 2020-02-03

**Authors:** Marianne Riou, Mégane Pizzimenti, Irina Enache, Anne Charloux, Mathieu Canuet, Emmanuel Andres, Samy Talha, Alain Meyer, Bernard Geny

**Affiliations:** 1 Unistra, Translational Medicine Federation of Strasbourg (FMTS), Faculty of Medicine, Team 3072 “Mitochondria, Oxidative Stress and Muscle Protection”, 11 rue Humann, 67000 Strasbourg, France; 2 Physiology and Functional Exploration Service, University Hospital of Strasbourg, 1 Place de l’Hôpital, 67091 Strasbourg CEDEX, France; 3 Pulmonology Service, University Hospital of Strasbourg, 1 place de l’Hôpital, 67091 Strasbourg CEDEX, France; 4 Internal Medicine, Diabete and Metabolic Diseases Service, University Hospital of Strasbourg, 1 place de l’Hôpital, 67091 Strasbourg CEDEX, France; emmanuel.andres@chru-strasbourg.fr

**Keywords:** pulmonary arterial hypertension, skeletal muscles, respiratory muscles, oxygen supply, catabolism, mitochondria, exercise

## Abstract

Pulmonary arterial hypertension (PAH) is a rare disease, which leads to the progressive loss and remodeling of the pulmonary vessels, right heart failure, and death. Different clinical presentations can be responsible for such a bad prognosis disease and the underlying mechanisms still need to be further examined. Importantly, skeletal and respiratory muscle abnormalities largely contribute to the decreased quality of life and exercise intolerance observed in patients with PAH. At the systemic level, impaired oxygen supply through reduced cardiac output and respiratory muscle dysfunctions, which potentially result in hypoxemia, is associated with altered muscles vascularization, inflammation, enhanced catabolic pathways, and impaired oxygen use through mitochondrial dysfunctions that are likely participate in PAH-related myopathy. Sharing new insights into the pathological mechanisms of PAH might help stimulate specific research areas, improving the treatment and quality of life of PAH patients. Indeed, many of these muscular impairments are reversible, strongly supporting the development of effective preventive and/or therapeutic approaches, including mitochondrial protection and exercise training.

## 1. Introduction

Pulmonary arterial hypertension (PAH) is a rare disease characterized by the pathologic remodeling of the pulmonary vasculature, which leads to right heart failure and death [1]. According to the last classification, pulmonary hypertension (PH) can be the consequence of different pathological conditions and is divided into five subgroups based on clinical presentation, hemodynamic characteristics, and therapeutic options [2]. Group 1 includes patients suffering from idiopathic or heritable PAH, drug and toxin-induced PAH, or PAH associated with connective tissue diseases. Group 2 PH is due to left heart diseases. Group 3 PH is due to respiratory diseases, including PH associated with chronic obstructive pulmonary disease (COPD) or pulmonary fibrosis. Group 4 includes chronic thromboembolic hypertension, and group 5 regroups PH patients with unclear multifactorial mechanisms.

Invasive hemodynamic assessment with right heart catheterization is requested to confirm the diagnosis of PH, and the definition of PH, in agreement with the European Society of Cardiology (ESC) and the European Respiratory Society (ERS) and the American college of cardiology (ACC) and American Heart Association (AHA) expert consensus on PH, was until recently based on resting mean pulmonary arterial pressure (PAP) ≥ 25 mm Hg [3,4]. At the 6th world symposium on PH, a modification of the hemodynamic definition of pre-capillary PH was proposed, including a mean PAP > 20 mmHg with pulmonary arterial wedge pressure (PAWP) ≤ 15 mmHg and pulmonary vascular resistance (PVR) ≥ 3 Wood Units [2]. 

To date, no clinically useful definition of exercise PH is available [3,5]. Currently, PAH remains a severe clinical condition despite the availability over the past 15 years of more than 10 drugs targeting three main pathways important in endothelial function (endothelin, nitric oxide, and prostacyclin pathways) [1]. Furthermore, PAH is often diagnosed at an advanced stage. Many attempts are ongoing to limit right heart alterations and to assess biomarkers with prognostic values, and there is growing evidence that PAH not only affects the pulmonary circulation but also exhibits systemic alterations, such as inappropriate angiogenesis, metabolic derangements, DNA damage, genetic mutations, impaired vasoreactivity, and muscles impairments [6,7,8].

All muscles are involved in PAH pathophysiology, particularly the right ventricular muscle that bears the prognosis of PAH, but peripheral muscle abnormalities, including skeletal and respiratory muscles largely contribute to the decreased quality of life and the exercise intolerance in PAH [9,10,11,12,13,14]. 

Since such peripheral muscles’ alterations appear to be reversible, better understanding the mechanisms underlying PAH-related muscle atrophy might be helpful for the development of effective preventive and/or therapeutic approaches. Accordingly, recent studies assessed exercise training in patients with PAH supporting an important benefit with improvement in the six-minute walk test or in the VO_2_ peak [15,16].

This review is focused on skeletal and respiratory muscle dysfunctions in PAH and, based on experimental and clinical data, examines whether such impairments might be related to the observed decrease in exercise intolerance. We particularly analyzed muscles mitochondrial function since mitochondria are the powerhouse of myocytes and are largely involved in both normal and abnormal muscle functions [17]. Sharing the new insights into the pathological mechanisms of PAH should stimulate specific research improving the treatment and quality of life of PAH patients. 

## 2. Skeletal and Respiratory Muscles in Normal Conditions 

The skeletal muscles represent more than one third of the total body mass, are responsible for 20% to 30% of resting oxygen consumption and receive more than 20% of the cardiac output (CO) [18,19]. The percentage of the CO diverted to respiratory and skeletal muscles increases significantly during maximal exercise, a situation where the O_2_ cost of breathing approaches 10% to 15% of the total VO_2_max (maximum O_2_ uptake) in healthy subjects [20].

Skeletal muscles are obviously involved in locomotion. However, their role extends far beyond and has been recently implied in the pathophysiology of various conditions, such as metabolic syndrome, immunology, cancer, memory, and depression [21,22,23,24]. In healthy men, muscle grip strength and exercise capacity have been shown to be predictors of all causes of mortality [25,26]. In the same line, a meta-analysis showed that a one kilo decrease of muscle grip strength is associated with an increase of 3% mortality in community dwelling people [27]. Thus, skeletal muscle is a crucial organ for healthy and patient populations. 

**Figure 1:**
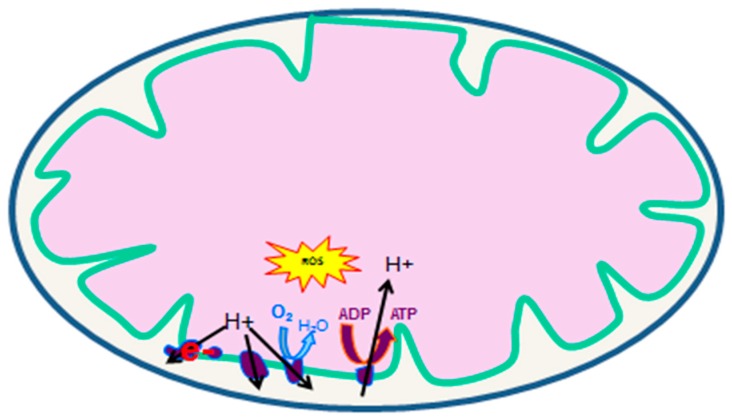
Mitochondrial respiratory chain complexes (Modified from [17]).

Particularly, muscle mitochondrial function is a critical factor modulating exercise capacities [28]. Indeed, mitochondria are the main source of cellular energy, coupling the oxidation of fatty acids and pyruvate to the production of a high amount of ATP by the electron transport chain. Briefly, the measurement of the oxygen consumption of muscle mitochondria is used to study the mitochondrial respiratory chain formed by five complexes located across the mitochondrial inner membrane. Free electrons are transferred from complex I to complexes II, III, and IV, thereby allowing complexes I, III, and IV to extrude protons from the matrix. The return of H + ions from the mitochondrial membrane interspace towards the matrix allows complex V (ATP synthase) to phosphorylate ADP into ATP (Figure 1). Mitochondria are also an important source of reactive oxygen species (mtROS) produced by the reaction between oxygen and a small proportion of electrons that leak mainly from Complex I and III of the electron transport chain [29,30]. ROS act as physiological signals that contribute to various cellular functions [31]. However, ROS in excess are detrimental, reacting with proteins, lipids, and DNA and leading to cell dysfunctions and/or apoptosis. Mitochondria are an important source of ROS, especially when malfunctioning, but are also vulnerable to oxidative damages that can further increase mtROS production [32]. Thus, the modulation of mitochondrial activity is crucial for skeletal muscle cells integrity and functions.

## 3. Skeletal and Respiratory Muscle Dysfunction During PAH

Experimental and clinical data are presented in Table 1 and Table 2. The physiopathology of the systemic myopathy observed in PAH is multifactorial. Currently, there is a lack of standardization between the clinical studies, including a generally small sample size of PAH patients and the fact that most of the clinical studies studying skeletal or respiratory muscle function in PAH have included idiopathic PAH patients (group 1 of the PH classification) and a majority of women (ages 45–60).

### 3.1. Skeletal Muscle Dysfunction During PAH (Figure 2)

#### 3.1.1. Catabolic Markers and Inflammation

Decreased muscle mass has been reported in idiopathic PAH patients [10]. Skeletal muscle atrophy contributes to muscle weakness and represents an imbalance and degradation of myofiber structural and contractile proteins lysis exceeding the muscle’s synthetic capacity [46]. Although protein degradation is achieved in eukaryotic cells through multiple proteolytic systems, research conducted in animal models has shown that ubiquitin-proteasome system (UPS)-mediated proteolysis is the predominant system activated in atrophying muscle [47]. Indeed, increased levels of both atrogin-1 and muscle RING finger protein (MuRF)-1 were measured in the atrophied quadriceps muscle of patients with PAH, suggesting that UPS-mediated proteolysis contributes to the skeletal muscle atrophy in these patients [10]. Moreover, UPS dysregulation is recognized to contribute to cardiovascular diseases [48]. Another study showed that experimental monocrotaline-induced PAH in rats results in the significant loss of skeletal muscle mass, accompanied by an increase in circulating and local catabolic markers of proteolysis, such as IL-1β CRP, myostatin, or MAFbx/atrogin 1 and protease activity [38]. Peripheral muscle weakness in patients with PAH is at least partly caused by sarcomeres’ dysfunction [43]. Many studies have demonstrated a reduction in maximal volitional and no volitional of the quadriceps as well as inspiratory muscles strength and endurance in PAH [9,11,12,34,39,41].

Other systemic factors could lead to skeletal muscle dysfunction in PAH. Inflammation thought to be a preponderant mechanism in pulmonary vascular remodeling in PAH could also be involved in PAH-related myopathy. Indeed, an elevated level of circulating pro-inflammatory cytokines, such as interleukin IL-1β, IL-2, IL-4, IL-5, IL-6, IL-8, IL-10, or tumor necrosis factor-α, are present in the PAH animal model or patients and could contribute to contractile dysfunctions in diaphragm or limb muscles [49,50,51].

#### 3.1.2. Impaired Oxygen Supply

**Figure 2:**
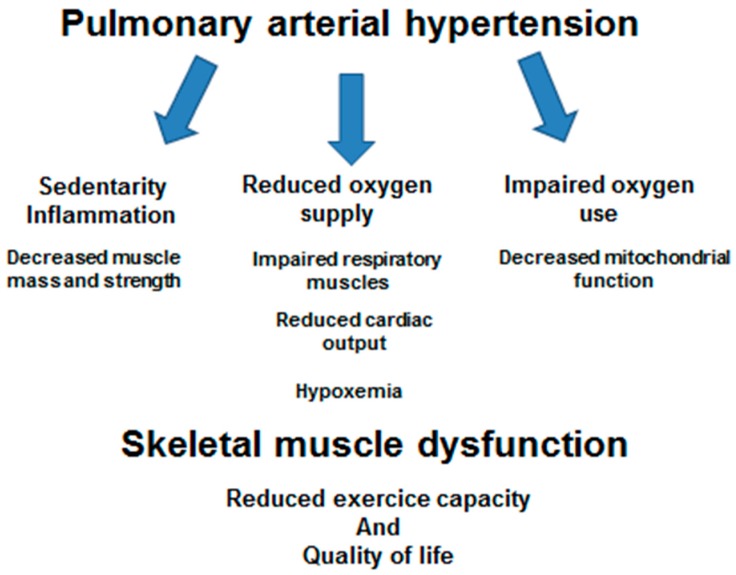
Mechanisms likely participating in pulmonary arterial hypertension-related skeletal muscle dysfunction.

Impaired oxygen supply to the peripheral muscle are likely due, at least partly, to decreased CO and impaired respiratory muscles, associated or not with hypoxemia. The impairment of peripheral muscle microcirculation and decreased capillary density within the skeletal muscle also influence exercise tolerance and quadriceps strength through impaired muscle oxygen supply in PAH [40,42]. Capillary rarefaction could be due to a down-regulation of microRNA-126. Indeed, the ectopic restoration of microRNA-126 increased capillary density and exercise capacity in a PAH animal model. Interestingly, the down-regulation of microRNA-126 is linked to VEGF signaling and/or mitochondrial energy metabolism and appeared to be specific to PAH muscles, since muscles from COPD patients had normal microRNA-126 levels [52,53]. Chronic hypoxemia is also involved in muscle dysfunction: the skeletal muscle microcirculation of patients with PAH appears to be impaired, as shown by low O_2_ saturation at the tissue level, which was measured by a near-infrared spectroscopy technique [40]. Moreover, abnormalities of the microvascular O_2_ delivery-to-utilization rate were found in woman with PAH [53]. Quadriceps muscle biopsies of PAH patients displayed higher phosphofructokinase/3-hydroxyacylcoA-dehydrogenase, suggesting an increased anaerobic metabolism [39]. This might potentially be linked to mitochondrial dysfunctions Figure 2.

#### 3.1.3. Impaired Oxygen Use: Mitochondrial Dysfunction

**Figure 3:**
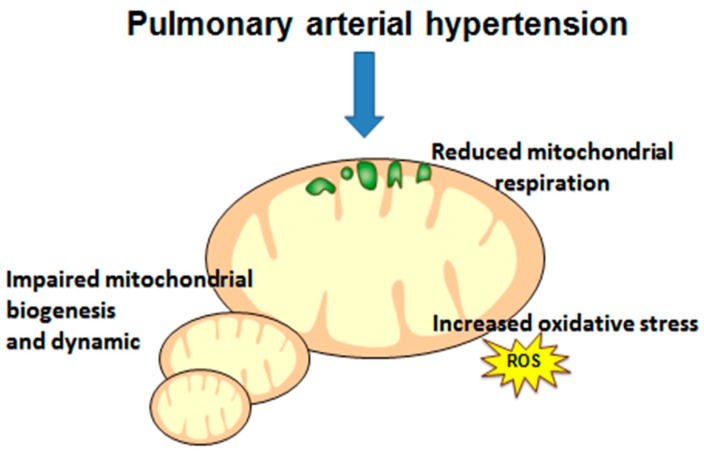
Mechanisms likely participating in pulmonary arterial hypertension-related mitochondrial dysfunctions in muscles.

As presented before, mitochondria are the main energy source of myocytes and their function needs oxygen to adapt to muscle demand. Mitochondrial hyperpolarization, endoplasmic reticulum stress, or enhanced anaerobic glycolysis contribute to PAH pathophysiology within the pulmonary artery smooth vessels, but the role of mitochondrial dysfunction in skeletal muscle is less well known [54]. However, mitochondrial dysfunction likely participates in skeletal muscle atrophy in patients with PAH (Figure 3). In monocrotaline-treated rats, abnormalities in mitochondrial biogenesis and respiration capacity have been documented in gastrocnemius muscle before right ventricular failure occurred [36]. In the same animal model, both mitochondrial quantity and quality were reduced in plantaris muscle [35]. Mitochondrial morphology depends upon the balance between fusion and fission, and recent studies have suggested that mitochondrial morphology influences muscle mass, but the underlying mechanisms are yet to be investigated. There is a decreased expression of proteins known to regulate mitochondrial fusion in skeletal muscle of PAH patients, which may additionally contribute to the development of muscle atrophy, as well as alterations in a protein regulating sarcoplasmic reticulum calcium sequestration, suggesting that impaired excitation–contraction coupling in the PAH muscle might contribute to muscle dysfunction in this population and to the limitation of exercise capacity [10]. On the other hand, it has been recently observed no primary mitochondrial oxidative phosphorylation dysfunction in skeletal muscles in idiopathic PAH patients. The authors suggested the possibility that impaired oxygen delivery to the mitochondria affects skeletal muscle bioenergetics during exercise [45] and that the reduced oxygen delivery could be due to central or peripheral factors such as reduced CO, decreased muscle capillary density, decreased angiogenesis, or abnormal peripheral microcirculation [37]. Nevertheless, confirming potential mitochondrial dysfunctions, experimental studies have also suggested a role for the accumulation of dysfunctional mitochondria due to the inability of protein quality control systems to efficiently eliminate damaged proteins in monocrotaline-induced PAH rats [38]. In eight PAH patients, a decreased expression of oxidative enzymes (pyruvate dehydrogenase) and an increased expression of glycolytic enzymes (lactate dehydrogenase activity) was observed [44]. Knowing that oxidative stress leads to lipid peroxidation, protein oxidation and DNA damage, it is likely that it also participates in PAH muscle weakness and fatigue, as supported by abnormal mitochondria morphology on electronic microscopy and by the fact that redox stress plays a pivotal role in PH-induced diaphragm weakness. 

### 3.2. Respiratory Muscle Dysfunction During PAH 

The diaphragm force deficit in PAH results from muscle wasting and intrinsic contractile dysfunction [34,55]. Many studies have shown a reduction in isometric force, which is normalized by the cross-sectional area in PAH patients and animals. In the study of Meyer et al., decreased respiratory muscle strength did not appear to be related to hemodynamics, blood gases, lung mechanics, exercise capacity, ventilatory efficiency, or even functional class, suggesting that other factors may play a role [11]. A combination of both systemic as well as local factors could contribute to the diaphragm muscle weakness [13]. A recent animal study suggests a role of redox stress in PAH-induced diaphragm dysfunction, leading to modifications of actin [56]. The administration of antioxidant treatment might prevent these changes. 

Interestingly, however, peripheral muscle and respiratory muscle activity greatly differ in PAH patients. Patients with PAH hyperventilate not only during exercise but also under resting conditions, leading to an overstimulation of the diaphragm contrary to the peripheral muscle activity, which generally decrease as physical activity declines with disease progression [57]. The overstimulation of diaphragm in PAH patients could explain its increased vulnerability to systemic factors but other factors are probably implicated. In monocrotaline-induced PAH rats, muscle fibers’ cross-sectional area and contractility were altered in the diaphragm muscle and not in the extensor digitorum longus muscle [55]. 

In humans, both expiratory and inspiratory muscle strength decreases have been observed, as well as peripheral muscle dysfunction [11], but time courses of skeletal and respiratory muscle dysfunction might differ. In addition to the first human study by Meyer et al., Kabitz et al. provided evidence that respiratory muscle strength is reduced in patients with PH and found that maximal inspiratory mouth and sniff trans diaphragmatic pressures were correlated with the 6-min walking distance [12]. Interestingly, another study ruled out the contribution of inspiratory muscle weakness/fatigue to the decreased inspiratory capacity observed during a maximal cardiopulmonary cycle exercise test, but this test was performed by selected young PAH patients [58].

## 4. Relationships between Peripheral Muscles Impairments, Decreased Exercise Performance, and Quality of Life in PAH Patients

PAH is a progressive disease. As PAP and PVR increase, right ventricle contractility improves initially to preserve CO and pulmonary and systemic perfusions. When PVR increases further, the right ventricle fails, leading to severe functional impairment and limited exercise capacity [5]. Indeed, PAH patients have a reduced capacity to augment CO during exercise, secondary to both smaller stroke volume and chronotropic impairment reducing the heart rate increase during exercise. Exercise intolerance is one of the main symptoms, patients complaining of leg fatigue and/or dyspnea and presenting with daily life reduced activities [59]. In addition to right heart dysfunction, abnormalities of both skeletal and respiratory musculature contribute to the pathology and clinical symptomatology of PAH [9,10,11,12,39]. 

Indeed, many observations have confirmed that exercise limitation in PAH is not merely due to pulmonary hemodynamic impairment. Skeletal and respiratory intrinsic muscles abnormalities, such as (1) reduced muscle strength, (2) decrease from “resistant” type I towards “more fatigable” type II fibers, (3) altered excitation–contraction coupling, (4) increased muscle protein degradation, (5) reduced muscle capillary density, and (6) impaired mitochondrial biogenesis/function occur independently of the severity of PAH [10,39]. PAH patients suffer from a systemic myopathy that presents an increased risk for a functional decline due to the loss of muscle function. Skeletal muscle alterations are associated with exercise intolerance in patients with PAH compared with control subjects: PAH patients have diminished VO_2_max, a lower anaerobic threshold, and a higher minute ventilation/CO_2_ [10]. Peripheral muscle inactivity might contribute to myopathy in PAH, which worsens as physical activity declines with disease progression. 

## 5. Therapeutics Approaches Targeting Muscle Dysfunction in PAH

As muscle dysfunction affects the exercise capacity and quality of life of PAH patients, therapeutic options that specifically improve skeletal muscle function are warranted. Pulmonary vasodilators interfering with the endothelin, nitric oxide, and prostacyclin pathways might improve oxygen supply to the muscle but are not sufficient to restore exercise capacity.

Exercise training (quadriceps muscle training and cycling) significantly improves muscle function and endurance capacity in PAH patients but its tolerability is limited in severely hemodynamically impaired patients with low CO [5]. Nevertheless, large improvements in a 6-min walk test distance were reported in several studies after exercise training [15,16,60]. A recent randomized controlled trial has shown that an 8-weeks exercise intervention including aerobic resistance and specific inspiratory muscle training significantly improves muscle power and other functional variables in PAH patients [61]. De Man et al. observed a significant improvement in exercise endurance and quadriceps muscle function and morphology through increased capillarization and oxidative enzyme activity after exercise training in PAH [62]. In addition, peripheral muscle improvement has been associated with decreased type II fiber proportion [63]. Moreover, PAH patients report a better quality of life and decreased sensation of dyspnea after inspiratory muscle training [60]. Beside the clinical effects, it has also been shown that exercise training may reduce inflammation and cell proliferation on a molecular level and may have a beneficial effect on the endothelial dysfunction [64,65]. Finally, exercise training improves mitochondrial respiratory capacity [66]. 

To date, a supervised and closely monitored exercise and respiratory training program in specialized clinics as an add-on to medical therapy is recommended for stable PAH patients (class IIa, level of evidence B) [3]. The ERS task force statement suggests that individually adjusted exercise training rehabilitation programs supervised by PAH expert centers and rehabilitation professionals are likely to be safe for patients with PH who are stable on medical therapy [67]. The involvement of a physiotherapist is a constant feature in all studies involving exercise programs in PH. Different approaches in exercise training modalities across countries are performed: in hospital start-of-exercise training followed by a secondary ambulatory part to allow closed supervision of exercise with heart rate and oxygen saturation monitoring at the beginning of the training [15,16,68,69,70] or outpatient (ambulatory) rehabilitation programs [60,61,62,71,72,73,74,75,76]. Training modalities are various according to the different studies, using bicycle ergometer, treadmill walking, cross-training, dumbbell training of distinct muscle groups, or breathing/respiratory muscle training [77]. In general, training intensity is low to moderate (50% of peak workload or 60% of maximal workload) and carefully monitored. Nevertheless, further randomized controlled trials are needed to confirm the data on the effect of exercise training on clinical parameters such as right ventricular function and hemodynamics, and to better understand the pathophysiological mechanisms by which exercise training is beneficial to patients with PAH [78] since only one study provides data on positive hemodynamic effect of rehabilitation in patients with PAH [79]. Based on the previous finding of a potential role of impaired oxygen delivery to the mitochondria, which results in skeletal muscle dysfunction in PAH, further research to improve peripheral factors that affect the oxygen transport pathway in PAH are needed [45].

A few experimental studies have suggested other potential treatment strategies to improve skeletal muscle function in PAH. Proteasome pathways inhibitors might improve skeletal muscle fiber size and contractile function [10]. Bortezomib has been studied in pulmonary arterial remodeling in rats [80,81]. Considering the capillary rarefaction in the skeletal muscle of patients with PAH, which is possibly caused by a downregulation of microRNA-126, therapeutic microRNA expression modulation could be interesting in these patients. Peripheral muscle weakness being partially caused by sarcomeres’ dysfunction, calcium sensitizers might be a therapeutic option [43]. Last but not least, treatments that targets mitochondrial dysfunction could be interesting to counter the skeletal muscle dysfunction in PAH. Indeed, many studies have demonstrated a benefit of antioxidant treatment, such as N-acetylcysteine on pulmonary vasculature in PAH-rats [82], and such therapy has been shown promise in experimental studies investigating peripheral arterial disease, which by many aspects resemble the PAH-related myopathy [83].

## 6. Conclusions 

Although PAH is a pulmonary vasculopathy, important cross-talk among lungs, heart, and skeletal muscle might contribute to the poor functional capacity and quality of life of patients with PAH. Skeletal muscle impairment appears to be a systemic manifestation of PAH, and this secondary myopathy deserves to be better investigated and treated. Many diseases, especially chronic diseases, result in skeletal muscle dysfunction, but whether they are the same in left heart failure, severe respiratory diseases, and in PAH deserves further studies. Some of the pathways involved are clearly similar. Accordingly, skeletal muscle mitochondrial dysfunction with reduced oxidative capacity has also been observed in patients with left heart failure, peripheral arterial disease and in patients suffering from COPD. On the other hand, comparing PAH and COPD, the down-regulation of microRNA-126 might be specific to PAH. Furthermore, whether the sex-related mitochondrial changes and exercise intolerance observed in left heart failure also occurs in PAH patients remain to be investigated [17,37,52,84,85].

To date, only studies on small numbers of patients were performed. In future, larger multicenter studies are warranted to determine the contribution of quadriceps muscle atrophy on reduced skeletal muscle function, the underlying pathophysiological mechanisms ranging from systemic (decreased oxygen supply) to local impairments (reduced mitochondrial oxidative capacity), and to adapt efficient exercise training protocols in PAH patients, potentially associating concentric and eccentric modalities. 

## Figures and Tables

**Table 1:** Experimental data supporting pulmonary artery hypertension-related skeletal and respiratory muscles alterations.

References	Animals	Animal Model	Type of Muscle	Main Results
Vescovo. 1998, Cardiovasc Res [33]	Rats	MCT injection	Peripheral (M. soleus, extensor digitorum longus)	- Fibers type I/II ratio
de Man. 2011, Am J Respir Crit Care Med [34]	Male Wistar rats	MCT injection	Respiratory (diaphragm)Peripheral(extensor digitorum longus/quadriceps)	- Diaphragm: twitch force, tetanic force, force-frequency of single muscle fibers - Smaller diaphragm cross-sectional area fiber
Wust. 2012, Am J Physiol Heart Circ Physiol [35]	Rats	MCT injection	plantaris	- Mitochondrial quantity and quality were reduced in plantaris muscle
Enache. 2013, Mol Cell Biochem [36]	Rats	MCT injection	Right/left ventriculeGastrocnemius	- Abnormalities of mitochondrial biogenesis and respiration capacity have been documented in gastrocnemius muscle biopsies before right ventricular failure occurred
Potus. 2014, Am J respir Crit Care Med [37]	Male Sprague-Dawley rats	MCT injectionintramuscular injection of antagomir-126 (anti-HAS-miR-126, 2 μg/quadriceps), every 4 days during a 2-week period	Peripheral	- Treated rats had decreased miR-126 expression in skeletal muscle in comparison to rats treated with vehicle - Decrease in microvessels density
Moreira-Gonçalves. 2015, Biochim Biophys Acta [38]	Male Wistar rats	MCT injection	collection of blood and gastrocnemius samples	- MCT group developed PAH, RV dysfunction, body and muscle wasting: reduction of 20% 16% and 30% on body weight, gastrocnemius mass and fiber cross sectional area, respectively - Muscle atrophy was associated with a decrease in type I MHC - Circulating (C reactive protein, myostatin and IL-1beta) and local catabolic markers (MAFbx/atrogin-1, protease activity) were increased in MCT animals - Mitochondria isolated from *gastrocnemius* of MCT animals showed decreased activity of ATP synthase, lower levels of Tfam, accumulation of oxidatively modified proteins together with reduced levels of paraplegin

MCT: monocrotaline.

**Table 2:** Clinical data supporting pulmonary artery hypertension-related skeletal and respiratory muscles impairments.

Reference	Population Number (*n* – PH/control)	Type of Muscle	Outcomes Measured	Main Results
Meyer. 2005, Eur Respir J [11]	46 26/20	Respiratory (diaphragm)	- Maximal PImax /PEmax measure	- PEmax/PImax in PAH patients
Bauer. 2007, Respir Med [9]	48 24/24	Peripheral (Forearm)	- Maximal isometric forearm muscle strength (handgrip)	- Isometric forearm muscle strength in IPAH patients - Direct correlation with 6MWD
Kabitz. 2008, Clin Sci Lond Engl [12]	62 31 (25 PAH/6 CTEPH)/31	Respiratory (diaphragm)	- PImax /PEmax measure (volitional twitch) - TWmo, TWdi (non volitional twitch)	- PEmax/PImax in PAH patients - in non-volitional twitch
Mainguy. 2010, Thorax [39]	20 10/10	Peripheral (limp muscle/quadriceps)	- Limb muscle cross-sectional area by CT scan - Quadriceps strength by maximal voluntary contraction and non-volitional magnetic stimulation of the femoral nerve - Quadriceps biopsy	- Fiber type I/II ratio - Maximal voluntary contraction, quadriceps twitch (non-volitional) - Muscular phosphofructokinase/3-hydroxyacyl-CoA-dehydrogenase ratio (capillary density) - Quadriceps strength correlated with VO_2_max
Dimopoulos. 2013, Respir Care [40]	32 8/8 (+ 16 with chronic heart failure)	Peripheral (thenar muscle)	- Tissue O_2_ saturation by near-infrared spectroscopy - After 3-min brachial artery occlusion	- Resting tissue O_2_ saturation in PAH patients (impairement of peripheral muscle microcirculation) - Slower reactive hyperemia time - Peripheral systemic vasocontriction
Breda. 2014, Plos One [41]	26 16/10	Peripheral (quadriceps)	- Exercise capacity - Body composition - CT area of limb muscle - Quadriceps biopsy	- Patients with PAH have a reduced percentage of lean body mass - Reduced respiratory muscle strength, reduced resistance and strength of quadriceps
Potus. 2014, Am J respir Crit Care Med [37]	40 20/20	Peripheral (quadriceps)	- Amount of microvessels - miR-126 expression	- Skeletal microcirculation loss - Impaired angiogenesis secondary to miR-126 downregulation
Batt J. 2014, Am J respir Cell Mol Biol [10]	22 12/10	Peripheral (Quadriceps/vastus lateralis)	- Quadriceps Femoris Muscle Cross-Sectional Area - Skeletal muscle biopsy - Muscle Immunohistochemistry and Morphometrics	- Quadriceps muscle cross-sectional area in patients with PAH - Type I/type II muscle fiber ratio - Cellular Signaling Networks Regulating Skeletal Muscle Mass Are Differentially Expressed in the Vastus Lateralis of Patients with PAH - Regulators of Mitochondrial Fusion, Mitofusin 2 and Mitofusin 1 in PAH - Ubiquitin-proteasome (UPS)-mediated proteolysis contributes to the loss of skeletal muscle mass in PAH
Malenfant. 2015, Med Sci Sports Exerc [42]	20 10/10		- Voluntary and nonvolitional dominant quadriceps muscle strength measures - Nondominant quadriceps biopsy	- PAH patients display capillary rarefaction within the skeletal muscle
Manders. 2015, Eur Respir J [43]	19 11/8	Peripheral (quadriceps)	- Sarcomeric function in permeabilized individual muscle fibres	- Muscle weakness in PAH patients is partly caused by sarcomeric dysfunction
Malenfant. 2015, J Mol Med. [44]	16 8/8		- Skeletal muscle proteomic profile	- Nine downregulated proteins (related to mitochondrial structure and function) and 10 upregulated proteins (glycolytic enzymes) in PAH skeletal muscle - Abnormal mitochondria morphology on electronic microscopy
Sithamparanathan. 2018, Pulm Circ [45]	9		- in vivo and in vitro assessment of mitochondrial function by ^31^P-magnetic resonance spectroscopy scans	- abnormal skeletal muscle bioenergetics by ^31^P-MRS during and after exercise

CTEPH: chronic thromboembolic hypertension; PAH: pulmonary arterial hypertension.
